# Impact of metal sulphides and partially reduced graphene oxide as a counter electrode on hybrid quantum dot sensitized solar cells performance

**DOI:** 10.1038/s41598-025-06209-0

**Published:** 2025-07-03

**Authors:** Sawsan A. Mahmoud, Asmaa F. Mansour, Moustafa E. Elsisi

**Affiliations:** 1 Egyptian Petroleum Research Institute, Nasr City, 11727 Cairo Egypt; 2 Faculty of Science, Physics Department, Zagazig University, Zagazig, Egypt

**Keywords:** Z_0.76_Co_0.24_S, Hybrid structure, Counter electrode, CdS QD_s_, SILAR method and ZnO, Energy science and technology, Materials science, Nanoscience and technology

## Abstract

In the present work, different photoanodes, namely Zinc Oxide (ZnO), Cadmium Oxide (CdO), and Titanium Oxide (TiO_2_) nanoparticles, were deposited on fluorine-doped tin oxide (FTO) by the blade-coating method. Different quantum dots (QD_s_) like Cadmium Sulphide (CdS) and Zinc Sulphide (ZnS) were deposited on the photoanode by using the successive ionic layer adsorption and reaction (SILAR) technique. In this concept, different metal sulphides such as NiS, Z_0.76_Co_0.24_S, CoNi_2_S_4_, and partially reduced graphene oxide (P-rGO) were coated on the FTO substrate, which act as counter electrodes. These metal sulphides were prepared in one-step hydrothermal synthesis and Partially reduced graphene oxide (P-rGO) was synthesized from the graphite powder according to the modified Hummers and Offman method for applying as a counter electrode in quantum dot sensitized solar cells QDSSC_s_. The structural, electrical, and optical properties of photoanodes and counter-electrodes were investigated. The J-V characteristics of quantum dot-sensitized solar cells and the other parameters were analyzed. The power conversion efficiency of different photoanodes and different counter electrodes was compared. As P-rGO is applied as a counter electrode in a hybrid quantum dot sensitized solar cells, it enhances a photovoltaic performance of the cell 6.8% improvement compared with Z_0.76_Co_0.24_S as a counter electrode. This is due to the good electrical properties of P-rGO. Due to the well separation between the light-generated electrons and the formed holes, the cell containing TiO_2_ QD_s_ with six layers of CdS QDs deposited on six layers of ZnS QDs as a hybrid structure and P-rGO as a counter electrode has the highest efficiency of 10.75% and the current density of 22.07 mA cm^− 2^ compared with other cells due to the wide band gap energy of TiO_2_ QD_s_ that absorbed a wide range of the spectrum. So, P-rGO is a good material to achieve the high-power conversion efficiency in this type of hybrid quantum dot sensitized solar cells (HQDSSC_s_).

## Introduction

Energy is the essential strategy that meets current demand without endangering the capacity of future generations. Global population growth and industrialization have led to a sharp rise in the energy needs. Long-term advantages will result from the advancement of infinitely cheap solar energy. Renewable energy sources can be used in a variety of ways to generate electricity, including geothermal, hydroelectric, solar, wind, and tidal energy^1^.

Solar energy, radiant light that improves sustainability, lowers pollution, and is easily harnessed worldwide, and is seen to be a good substitute source to lessen reliance on fossil fuels. It is also the only method to generate efficient power^2–4^.

The market for non-silicon solar cells, like perovskite and dye-sensitive solar cells (DSSCs), is expanding quickly. A modified version of DSSC, known as solar cells with quantum dots as sensitizers (QDSSCs), can absorb more light/unit area than a typical silicon-based one^5–8^.

Due to its high-power conversion efficiency, ease of manufacture, and possible low cost, DSSCs have attracted much attention in the previous ten years^9^. Despite the tremendous efforts of numerous researchers to improve the efficiency of DSSCs, power conversion efficiencies higher than 12% have not been attained through the creation and modification of novel, difficult dyes^10–12^. Improve the conversion rate of electricity through the harvesting of light energy in the visible and infrared spectrum, narrow-band QD sensitizers, such as CdSe^13–15^, CdS^16^, CdTe^17^, PbS^18^, and Ag_2_S^18^, have taken the role of dyes.

This enables a multiple exciton generation (MEG) effect, a high extinction coefficient, a quantum confinement effect, and a tunable band structure. Among the various parts that comprise QDSSCs an inorganic solar cell devices are photoanodes, sensitizers, counter electrodes, and electrolytes, all of which are essential for generating the required quantity of electrical energy^19–21^. Employing mesoporous TiO_2_ layers, it was demonstrated that mesoporous oxide coatings could help extend the electron diffusion length^22^. For the best possible construction of solar cells, carrier mobility should also be considered besides diffusion length management. It was discovered that, in the microsecond time scale, the electron and hole mobilities were about equal and stayed high^23^.

However, if the injected electrons’ mobility in the TiO_2_ layer is slower than that of perovskite, its efficiency can be declining in the mesoscopic structure. An excessive photovoltaic efficiency requires a proper oxide layer design with high electron mobility. Among the metal oxide layers that have been researched for mesoscopically structured perovskite solar cells electron injection. Because ZnO has a superior electron mobility to TiO_2_, it has been proven to be one of the best energy collection devices^24,25^. ZnO is an alternative to TiO_2_ due to its suitable energy levels and good electron transport properties^26,27^.

The use of platinum as the counter electrode (CE) in QDSSCs is one factor driving up the device cost; on the international market, platinum is nearly as expensive as gold. Additionally, the Pt electrode cannot be used on flexible plastic boards due to the high sintering process (> 350 °C). Pt CE sustainability is also a problem because the I^−^/I^− 3^ redox pair is known to dissolve them and alter their valence state^28,29^.

For this reason, it’s critical to search for less expensive Pt substitutes, which have significantly higher production efficiency. Carbon materials^30–33^, polymers^34,35^, composites^36,37^, metal oxides^38,39^, and metal sulphides^40–42^ are a few of the suggested Pt-free substitutes. Because of their high catalytic effectiveness and cheaper production costs, metal sulphides, including binary and ternary metal components are the most appealing^41^. Its redox characteristics and multifunctionality could be improved by the use of a multi-cation component. NiCo_2_S_4_^43^, CoNi_2_S_4_^44^, CoMoS_4_ and NiMoS_4_^45^ are a few of the dual metallic sulphides. Graphene oxide (GO), which is produced by the chemical oxidation of graphite, has attracted considerable attention because of its industrial potential for the mass production of graphene powders (GPs) via chemical and thermal reduction. Further, GO is compatible with wet-based or polymer-based coating processes; therefore, a variety of pure or composite GP films can be produced. Recently, chemically- and thermally reduced GP films have been used as flexible electrodes and transparent electrodes in organic solar cells and dye-sensitized solar cells (DSSCs). However, the solar power conversion efficiencies of DSSCs and organic solar cells with conductive GP films are low: 0.84% for DSSCs and 1.1% for organic solar cells^58^. Graphene widely attracts researchers due to its low cost, suitable electrical conductivity, large surface area, and good electrochemical stability. Graphene is a two-dimensional structure of graphite, an allotrope of carbon, and an ultrathin carbon sheet. These properties have motivated the use of graphene as an electronic promoter in transparent electrodes for DSSC_s_. The graphene is used instead of expensive materials in electrochemical devices without compromising application performance. Graphene exhibits faster electron-transfer kinetics and better electrocatalytic properties. Graphene can form an efficient conductive network in the counter electrode^59^.

This work aims to study the effect of metal sulphide (NiS), binary metal sulphide (Z_0.76_Co_0.24_S), spinel sulphide (CoNi_2_S_4_) and partially reduced graphene oxide (P-rGO) as counter electrodes on the photovoltaic performance of the hybrid structure ZnS QD_s_ and CdS QD_s_ sensitized solar cells by applying different photoanodes as ZnO, CdO and TiO_2_ nanostructures.

## Experimental methods

### Materials

(Zinc Acetate Zn (CH_3_COO) _2_ (97%, Bio Chem), Sodium Sulfide Na_2_S (98%, Alpha chemical), Ethanol absolute (99%, Bio Chem), Triton -X100 (97%, Aldrich), Titanium (IV) isopropoxide (97%, Aldrich), Sulfure Powder (Adwic), Potasium Chloride (Advent), FTO (Fluorine-dopped SnO_2_) conductive glass (Aldrich), Nickle Chloride (NiCl_2_.6H_2_O), Cobalt Nitrate (Co (NO_3_)_2_) and Cupper Chloride (CuCl_2_.2H_2_O) from Adwic).

### Synthesis of zinc oxide nanoparticle (ZnO)

ZnO was prepared by the simple precipitation method as follows: 30 g of [Zn (O_2_CCH_3_)_2_(H_2_O)_2_] was dissolved in deionized water, then 0.1 M of NaOH solution was added dropwise until the pH = 9 is reached, while stirring vigorously for 1 h. To get rid of any ions that might have remained in the end product, the precipitate was washed with distilled water several times followed by the washing with ethanol to break down the agglomeration, and it was eventually dried for 24 h at 80 °C in a drying furnace, then the final product was calcined at 600 °C for two hrs.

### Synthesis of cadmium oxide nanoparticle (CdO)

**Fig. 1:**
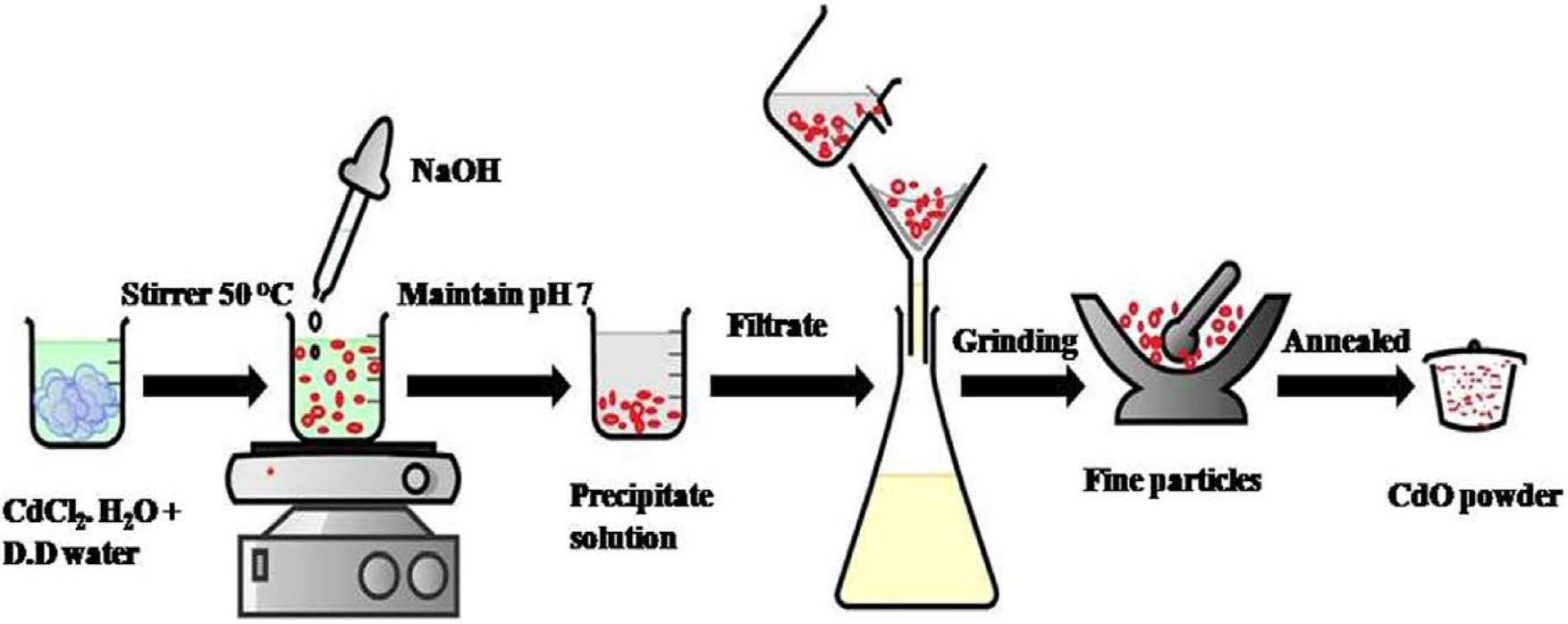
Schematic steps for the synthesis of CdO NP_s_ by the precipitation method.

NaOH solution was added dropwise to a solution of 20 g of cadmium chloride monohydrate [CdCl_2_.H_2_O] dissolved in DD water, while being continuously stirred until the pH reached 7. After that, the precipitate was constantly swirled for 4 h. The generated precipitate was allowed to settle before being repeatedly cleaned with DI water and dried for 24 h. at 80 °C. As a final step, the resultant powder sample was annealed for 2 h at 600 °C^48^ (Fig. 1).

### Synthesis of metal sulphide (NiS)

Using a straightforward in-situ hydrothermal approach, the counter electrodes based on binary metal sulfide (NiS) were created. Typically, 30 ml of distilled water was mixed with 0.15 M of nickel chloride hexahydrate (NiCl_2_·6H_2_O), 1 M of thiourea (SC(NH_2_)_2_), and 0.05 mL of ethylenediamine using magnetic stirring for 30 min to generate NiS nanoparticles. After that, the mixture had been transferred to a 100 mL stainless steel autoclave with a Teflon lining heated at 180 °C for 24 h. The produced NiS powder was cooled to room temperature, and then it was cleaned with ethanol, repeatedly with distilled water, and dried at 60 °C in the air^40^.

### Synthesis of binary metal sulphide (CoNi_2_S_4_ and Zn_0.76_Co_0.24_S)

The binary metal sul phide (CoNi_2_S_4_ and Zn_0.76_Co _0.24_S)-based counter electrodes were created using a straightforward in-situ hydrothermal approach. Cobalt nitrate [Co (NO_3_)_2_], nickel chloride hexahydrate (NiCl_2_·6H_2_O), thiourea (CH_4_.N_2_S), and 0.05 mL of ethylenediamine were dissolved in 30 mL of distilled water by magnetic stirring for 30 min to generate CoNi_2_S_4_ nanoparticles. Subsequently, the mixture was transferred to a 100 ml stainless-steel autoclave with a Teflon lining. For a duration of 24 h at 180 °C. The produced CoNi_2_S_4_ nanoparticle was cooled to ambient temperature, and then it was dried in the air at 60 °C, rinsed with ethanol, and repeatedly distilled. Zn_0.76_Co_0.24_S nanoparticles were made using a similar process, commencing with 0.5 mL of zinc chloride (ZnCl_2_), 0.1 M of cobalt chloride hexahydrate (CoCl_2_·6H_2_O), 1.2 mL of thiourea (CH_4_.N_2_S), and 0.05 mL of ethylenediamine^40^.

### Preparation of partially reduced-graphene oxide (R-GO)

Partially-reduced graphene oxide (P-rGO) was synthesized from the graphite powder according to the modified Hummers and Offman method. In a solution of 50 ml of concentrated H_2_SO_4_ and 50 ml of concentrated HNO_3_, the extra pure graphite powder (2.0 g) was pre-oxidized by slowly adding it to the mixture and stirring at 80 °C for 4 h. The mixture was cooled to room temperature and washed with de-ionized water until the pH value was neutral (equal to 7.0), followed by drying at 40 °C overnight. The resultant pre-oxidized graphite was dispersed into concentrated H_2_SO_4_ in a cold reaction vessel, which was kept in an ice bath and stirred. 10 g of KMnO_4_ was slowly added to it. During the addition, the temperature was kept below 10 °C. The mixture was stirred at 35 °C for 2 h until the solution became gelled and turned a brownish gray. Then 250 ml of de-ionized water was added and the temperature was raised to 100 °C for 15 min, followed by adding 700 ml of de-ionized water and 30 ml of H_2_O_2_ to the mixture, which was stirred for 1 h. The solid products were composed from the solution after 12 h and washed with 5% HCl until sulphate ions were no longer detectable with BaCl_2_. Then the solid products were redispersed in the deionized water five times to eliminate the impurities. Finally, the resultant sediment was dried at 60oC for 4 h in an oven to yield the partially reduced graphene oxide P-r (GO)^57^.

### Fabrication of TiO_2_, CdO, and ZnO nanoparticles working-electrodes

A 0.12 mm thick nanoparticle films were made from TiO_2_, CdO, and ZnO powders. The doctor-blading approach was used to deposit the TiO_2_, CdO, and ZnO NP samples onto conductive fluorine-tin-oxide (FTO) glass substrates. 0.2 g of TiO_2_ powder was dispersed in an ethanol solution, and the same was done for the CdO and ZnO NPs. The FTO substrates’ surfaces were ultrasonically cleaned for 15 min before the distribution of the layer. After that, the substrates were air-dried. Initially, the powder was scattered in Triton-X100 and ethanol.  To regulate the film’s thickness, Scotch tape was used to cover the FTO boundaries of each substrate. The pending powder was put as drops in the middle of the substrate and prevalence to build a thick layer. The film was calcined at 300 °C for 5 min after being dried in the air for 30 min^46^.

### Preparation of photoelectrode with hybrid structures (CdS/ZnS)

**Fig. 2:**
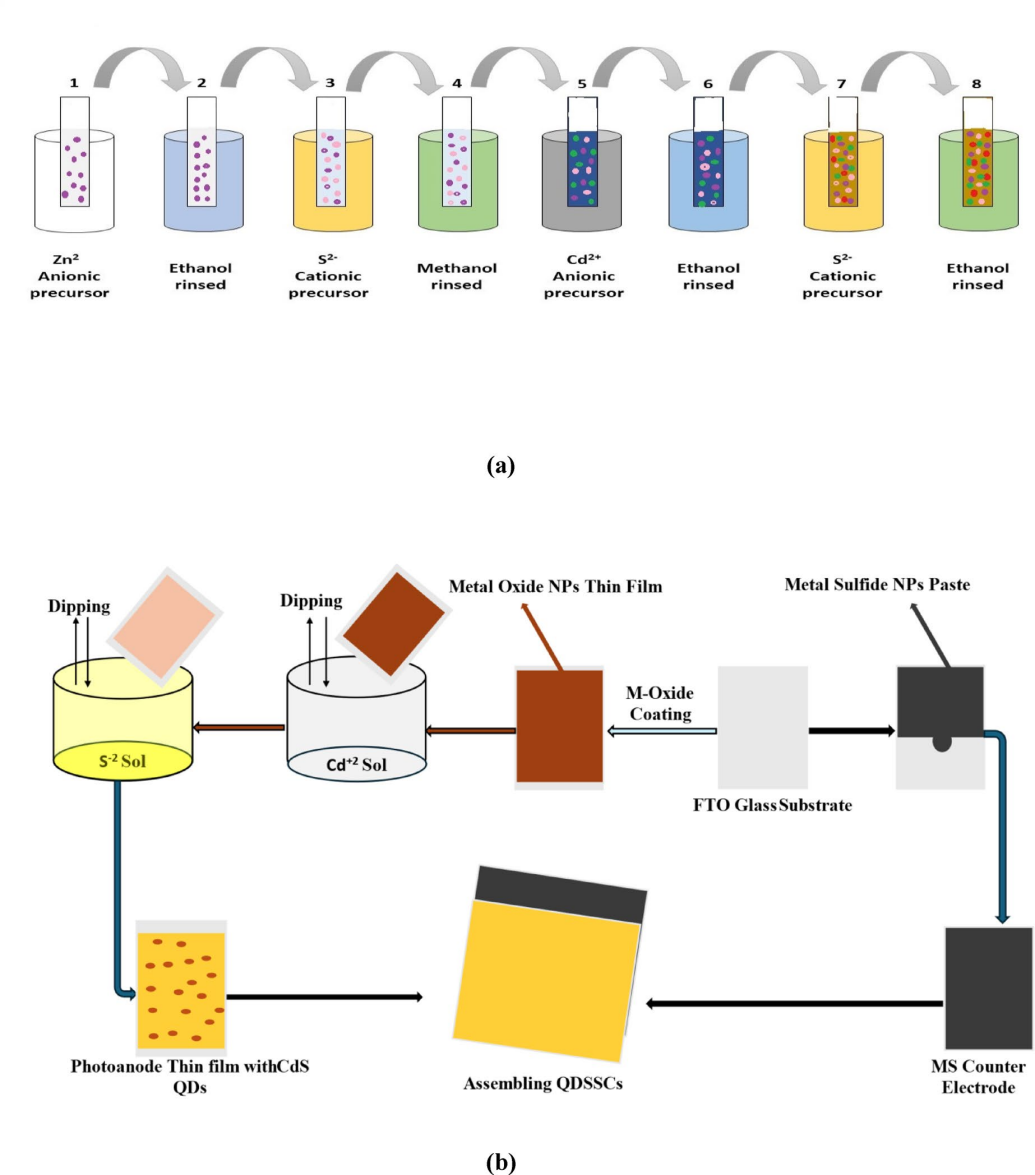
(**a**) Schematic representation of the SILAR process. (**b**) A general scheme representation of QDSSCs.

The CdS QDs, which function as light absorbers, were used to sensitize the photoelectrode using the Successive Ionic Layer Adsorption and Reaction (SILAR) technique. The semiconductor film was submerged for two min as a Cd^2+^ source in an aqueous solution containing 0.2 M Cadmium Nitrate (CdNO_3_)_2_, washed in ethanol to eliminate any excess ions, and then dried for one min at 60 °C on a hot plate. Then, the film was submerged for an additional two minutes in an aqueous solution containing 0.2 M Na_2_S to enable S^2−^to react with the Cd^2+^ that had already been adsorbed and create CdS QDs. After swilling the film in methanol to remove the weakly attached S^2−^ions, the film was dried for 1 min at 60 °C (Fig. 2a).

ZnS QD growth is conducted similarly to SILAR for preparing the hybrid structures of ZnS/CdS QDs. First, the semiconductor film was indigestible for 2 min in an aqueous solution containing 0.2 M Zn (CH_3_COO)_2_, rinsed with ethanol to remove any remaining surplus ions, and then dried for 1 min at 60 ºC on a hot plate. After that, the film was submerged for an additional 2 min in an aqueous solution containing 0.2 M Na_2_S to allow S^2−^to react with the Zn^2+^ that had already been adsorbed, forming ZnS QDs. After swilling the film in methanol to remove the weakly attached S^2−^ions, the film was dried for 1 min at 60 °C.

Subsequently, the ZnS QD layer and a semiconductor were immersed. For 2 min, a 2 M cadmium nitrate (Cd (NO_3_)_2_) aqueous solution was utilized as a source of Cd^2+^. After allowing S^2−^to react with the pre-adsorbed Cd^2+^ for an additional two minutes, the film was dipped into an aqueous solution containing 0.2 M Na_2_S. This process resulted in the production of hybrid structures consisting of CdS QDs and ZnS QDs. After washing the film in methanol to remove the loosely attached S^2−^ions, it was dried for 1 min at 60 °C. To improve the crystallinity of QDs, the layers with various quantum dot depositions were calcined for 5 min at 300 °C^47^.

### Preparation of electrolyte

An electrolyte made of polysulfide was produced and employed. The electrolyte consists of a mixture of methanol and deionized water in a volume ratio of 7:3, including 2 M Na_2_S, 2 M sulfur powder, and 0.2 M KCl. Between the top of the anode coated with QDs and the counter electrode, the electrolyte was placed^46^.

### Assembling the cds QDs sensitized solar cell

After the two electrodes—the photo and counter electrodes—were attached to each other using two clips and facing each other, drops of electrolyte solution could be placed in the plate corners. The two warp clips close, rotate, and turn off when in position. In the space between the electrodes, the electrolyte was placed by the capillary action (Fig. 2b). Light was directed towards the CdS QDs and CdS/ZnS adsorbed onto the film electrodes nanoparticles within the solar cell by exposing each solar cell device to light from the light source.

### Photoelectrochemical efficiency

The solar cell was tested with a 100 mW/cm^2^ light output under strained sun illumination. In addition to measuring cell efficiency, photocell software measures the J-V curve for each solar cell, which is recorded in light and dark conditions. Using the J-V characteristics as a function of incident light intensity, the open-circuit voltage (V_oc_), maximum voltage point (V_max_), maximum current density point (J_max_), and short-circuit current density (J_sc_) were calculated.

### Methods of analysis

With a Pan Analytical Model X ‘Pert Pro equipped with CuKα radiation (α = 0.1542 nm), a general area detector, and a Ni-filter, X-ray diffraction patterns were reported. The 40-mA emission current and 40 kV accelerating voltage were used. The diffractograms were measured between 0.5 and 70° in the 2θ range. The Fourier transform infrared spectroscopy (FT-IR) of the prepared samples was analyzed using the KBr approach employed by the Nicolet Is-10 FT-IR spectrophotometer (Thermo Fisher Scientific. The composition, shape, and size of the material are examined using a Field Emission Scanning Electron Microscope (FE-SEM). A JSM-7500F electron microscope operating at 30 kV acceleration voltage was recorded. Optical absorption spectra of the Samples were examined using Ultraviolet-Visible absorption spectroscopy (Spectro UV-Vis 2800, United States). Dielectric studies of TiO_2_ NPs, CdO and ZnO NPs as a working electrode and NiS, Zn_0.76_Co_0.24_S and CoNi_2_S_4_ as a counter electrode were executed as thin films on conducting glass (FTO) of dimension (6.25 cm^2^ surface area and a 0.07 mm thickness) for every sample to serve as electrodes during the measurements by a standard two-probe technique using an impedance analyzer (IM3570, Japan).

### Results and discussion

#### XRD analysis

**Fig. 3:**
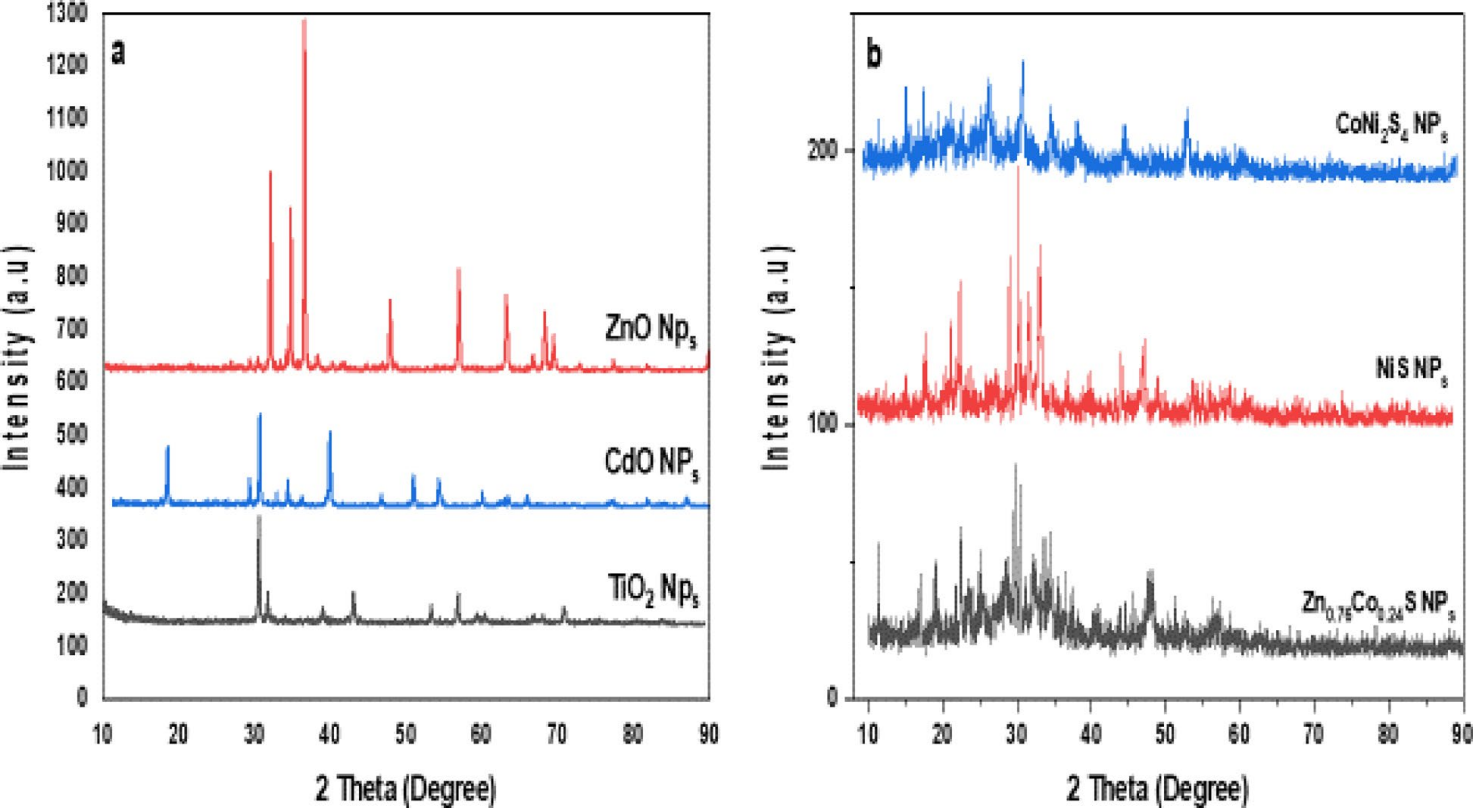
XRD spectra of (**a**) TiO_2_, CdO and ZnO, (**b**) NiS, CoNi_2_S_4_ and Zn_0.76_Co_0.24_S nanostructures.

Figure 3a depicts the X-ray diffraction pattern (XRD) for the synthesized CdO, TiO_2_, and ZnO nanoparticles. This XRD spectrum of the CdO indicates the number of strong diffraction peaks at the angles such as 18.5, 29.27, 30.65, 34.32, 39.94, 46.68, 50.92, 54.34, 60.04, 65.95, and 86.74, the corresponding reflection peaks belong to the (hkl) value such as (110), (200), (220), (311), (321), (322), (211), (200), (210), (220) and (222) [JCPDS 05–0640]^49^. These indexed peaks confirm the simple cubic structure. The primary peak at 33.1 denotes the CdO nanoparticles’ (110) crystallographic plane, which primarily characterizes the particles’ good crystalline structure.

The sharp diffraction peaks of pure ZnO NPs at 31.76, 34.39, 36.24, 47.54, 56.59, 62.82, 66.28, 67.89, 69.08 and 76.99 are indexed to (1 0 0), (0 0 2), (1 0 1), (1 0 2), (1 1 0), (1 0 3), (2 0 0), (1 1 2), (2 0 1) and (2 0 2) planes respectively. Figure 1 confirms that all the diffraction peaks belong to the hexagonal ZnO wurtzite structure (JCPDS no. 36-1451). All diffraction peaks for the TiO_2_ were congruent with the tetragonal structure TiO_2_ anatase phase and all diffraction peaks were in good agreement with JCPDS No. 21-1272. The peaks of diffraction angle existing at 2θ = 25.210, 37.600, 47.940, 51.450, 53.920, 55.010, 62.590, and 65.420 are corresponding to the (101), (004), (200), (105), (211), (204), (116) and (215) lattice planes respectively.

Figure 3b Shows the diffraction peaks of NiS at 19.2, 22.4, 23.64, 30.35, 31.62, 32.98, 34.6, 38.02, 45.34 and 48.42 indexed as (1 0 1), (3 0 0), (0 2 1), (2 2 0), (2 1 1), (1 3 1), (4 1 0), (4 0 1), (3 3 0) and (0 1 2) planes are well matched with rhombohedral structure of NiS (JCPDS no: 12–0041)^40^. The XRD pattern of CoNi_2_S_4_ shows diffraction peaks at 15.91, 18.23, 26.88, 31.68, 35.43, 38.85, 45.29, 53.66 58.73 and 61.28 corresponds to (2 2 0), (3 1 1), (4 0 0), (4 2 2), (5 1 1), (4 4 0), (5 3 3), (4 4 4), (6 4 2) and (7 3 1) planes confirm the formation of cubic structure (JCPDS no: 24–0334). The diffraction pattern of Zn_0.76_Co_0.24_S shows peaks corresponding to (1 1 1), (2 0 0) (2 2 0), (3 1 1), (4 0 0), and (3 3 1) planes which are well-matched with a pure cubic phase of Zn_0.76_Co_0.24_S (JCPDS no: 47-1656)^40^. XRD analysis revealed the formation of phase pure binary NiS and ternary CoNi_2_S_4_ and Zn_0.76_Co_0.24_S compounds by the careful control of synthesis conditions.

In addition, the average crystallite size (D) of spinels aluminates nanostructures can be predestined from the full width at the half maximum of the strongest diffraction peak by applying the Debye–Scherrer equation^50^; D = 0 0.9 λ/βcos(θ) Where D is the average crystallite size in Å, k 0.9 is the shape factor, λ is the wavelength of X-ray Cu Kα radiation (1.5406 Å), θ is the Bragg diffraction angle, β is the full width at half maximum (FWHM), of the respective diffraction peak. The crystalline sizes are predicted as 9.11 nm, 12.35 nm, 7.23 nm, 18.3 nm, 3.66 nm, and 10.15 nm for CdO, ZnO, TiO_2_, NiS, CoNi_2_S_4_, and Zn_0.76_Co_0.24_S, respectively.

### FTIR analysis

**Fig. 4:**
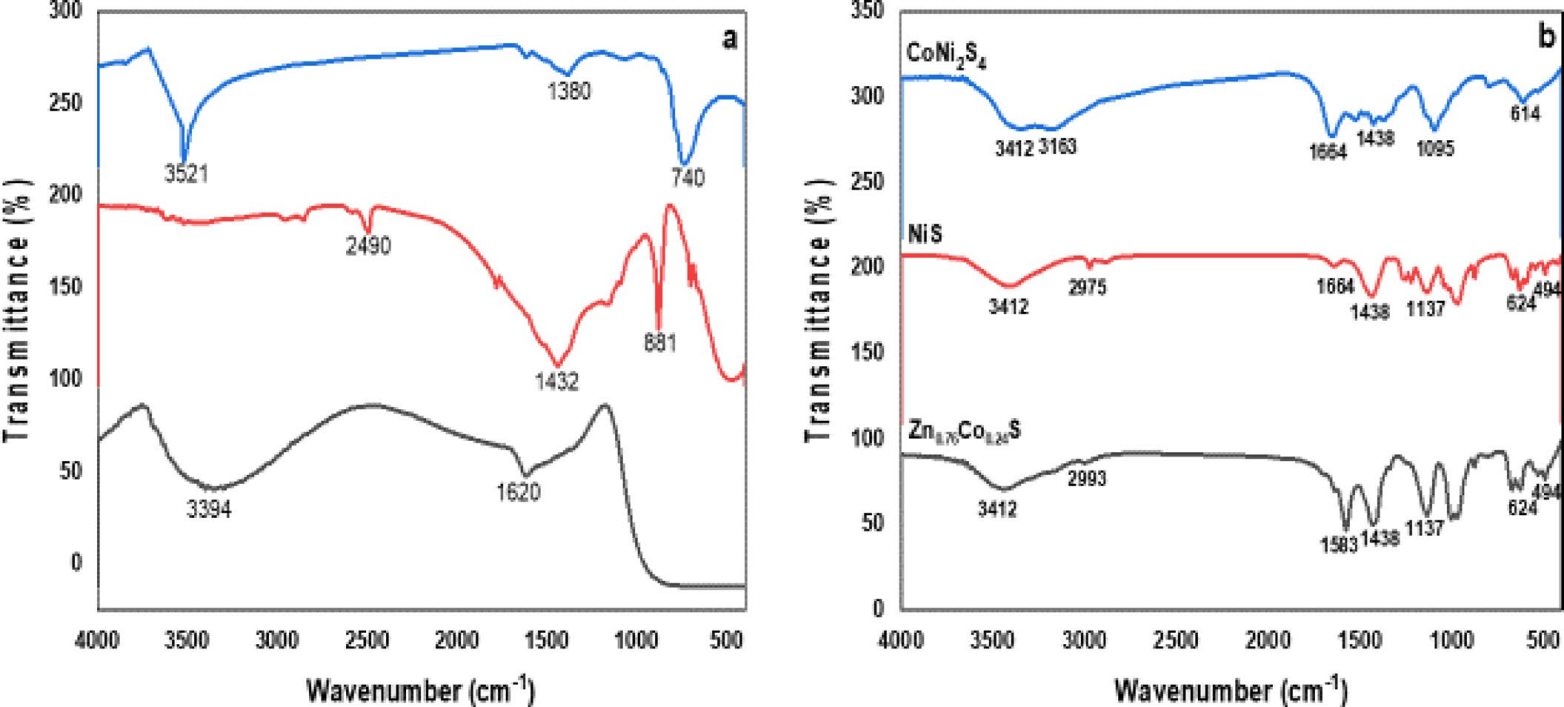
FTIR spectra of (**a**) TiO_2_, CdO and ZnO, (**b**) NiS, CoNi_2_S_4_ and Zn_0.76_Co_0.24_S nanostructures.

The synthesized nanoparticles’ FT-IR spectra is displayed in Fig. 4a. The transmittance spectra of CdO nanoparticles shows three main bands at 3413 cm^− 1^, 1386 cm^− 1^, 493 cm^− 1^, and 531 cm^− 1^. The O–H stretching vibration of water molecules is the cause of the stronger band seen at 3414 cm^− 1^^7^. The C–H stretching mode of vibration is responsible for assigning the sharper peak in the 1386 cm^− 1^ range^6^. The presence of oxygen and cadmium in this area is responsible for the metal bands at 493 and 531 cm^− 1^. The majority of the metal compounds should be found in this area^17^. This is the CO_2_ Peak of the FT-IR instrument’s inner atmosphere carbon dioxide.

FTIR spectrum range of TiO_2_ QD curves after 400 °C calcination. The presence of TiO_2_ as a crystalline phase is confirmed by the absorption band seen in the 400 cm^− 1^ to 800 cm^− 1^ range, which is linked to the bending vibration (O-Ti-O) links in the TiO_2_ lattice^51–53^. The distinctive bending vibration of the − OH group is seen by the strong peak at 1620 cm^− 1^^52–54^. The interaction between the hydroxyl group of water molecules and the surface of TiO_2_ is responsible for the broad absorption peak found in the range 3200 to 3800 cm^− 1^^53–56^. As a result of the sample’s water molecules being removed through calcination, the strength of the higher absorption bands diminishes.

All of the peaks in the spectrum for the ZnO sample had relatively low intensities. The (C-H) peaks are located between 2850 and 2966 cm^− 1^. The peaks corresponding to the hydroxyl stretching vibration (OH) are located between 3060 and 3826 cm^− 1^. The (C = O) stretch of hydrogen-bonded carboxylic acid groups is found in the carbonyl stretching area between 1680 and 1710 cm^− 1^, while that of free non-hydrogen-bonded carboxylic acid groups is found between 1710 and 1760 cm^− 1^ (Pawsey et al., 57). Typically, the carboxylic acid dimer’s (C = O) stretch is located between 1700 and 1720 cm^− 1^. ZnO spectrum demonstrating (Zn-O) presence at 433 and 1385 cm^− 1^. Sharp bonding O-H bond alcohol sites in monomeric form are responsible for the band at 3412 cm^− 1^. The C–O and C = O stretching modes, which correspond to the fingerprint region and functional group region of the NiS at 1137 cm-1 in Fig. 4b, were linked to the peaks at 1438 cm^− 1^ and 1664 cm^− 1^.

Sharp bonding O-H bond alcohol sites in monomeric form are responsible for the band at 3412 cm^− 1^. The C–O and C = O stretching modes, which represent the fingerprint area and functional group region of the CoNi_2_S_4_ at 1095 cm^− 1^ illustrated in Fig. 4b, were ascribed to the peaks at 1438 cm^− 1^ and 1664 cm^− 1^.

Sharp bonding O-H bond alcohol sites in monomeric form are responsible for the band at 3412 cm^− 1^. The C–O and C = O stretching modes, which correspond to the fingerprint area and functional group region of the Zn_0.76_Co_0.24_S at 624 cm^− 1^, were identified as the source of the peaks at 1438 cm^− 1^ and 1664 cm^− 1^.

### Morphological analysis: FESEM and EDX analysis

**Fig. 5:**
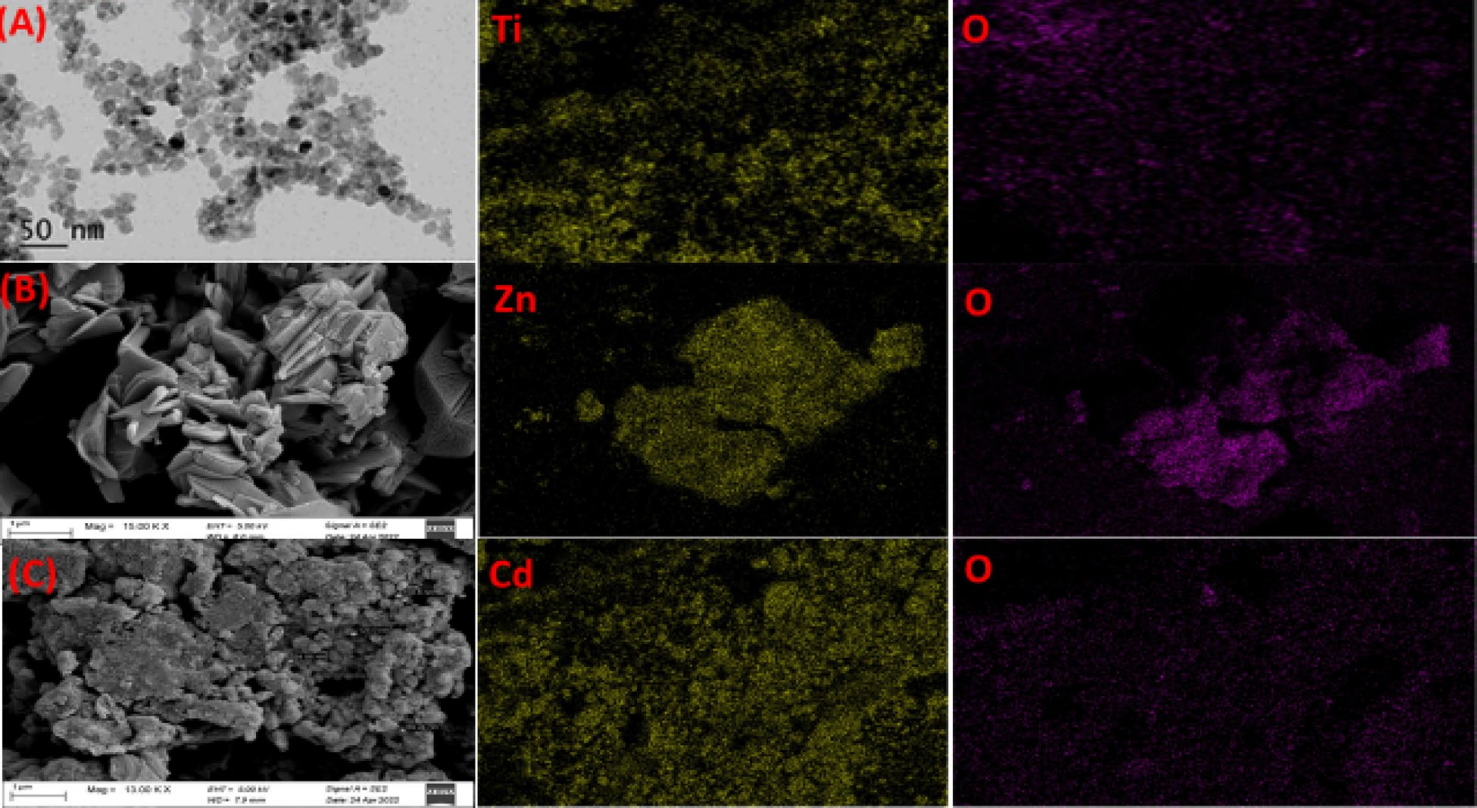
FE-SEM of (**A**) TiO_2_, (**B**) CdO and (**C**) ZnO nanostructures.

The element distribution of compounds for CdO, TiO_2_, and ZnO nanostructures is displayed in Fig. 5, along with the FESEM image. Every sample exhibits uniform surface morphology. The examination indicates that the cone-shaped granules, with an average diameter of 88.92 nm, form pure CdO phase and become significantly closer and packed firmly with aggregation when annealed at 600 °C, as shown in Fig. 5C. The energy dispersive X-ray analysis (EDX) of the CdO powder that was annealed at 600 °C is displayed in Fig. 5, confirming the presence of oxygen and cadmium. The FE-SEM images of TiO_2_ QDs annealed at 400 °C, as shown in Fig. 5A, reveal that the particles are nearly irregular in shape and homogenous in composition. The average diameter is 8.43 nm, and it is discovered that the particle size has grown and is strongly agglomerated.

**Fig. 6:**
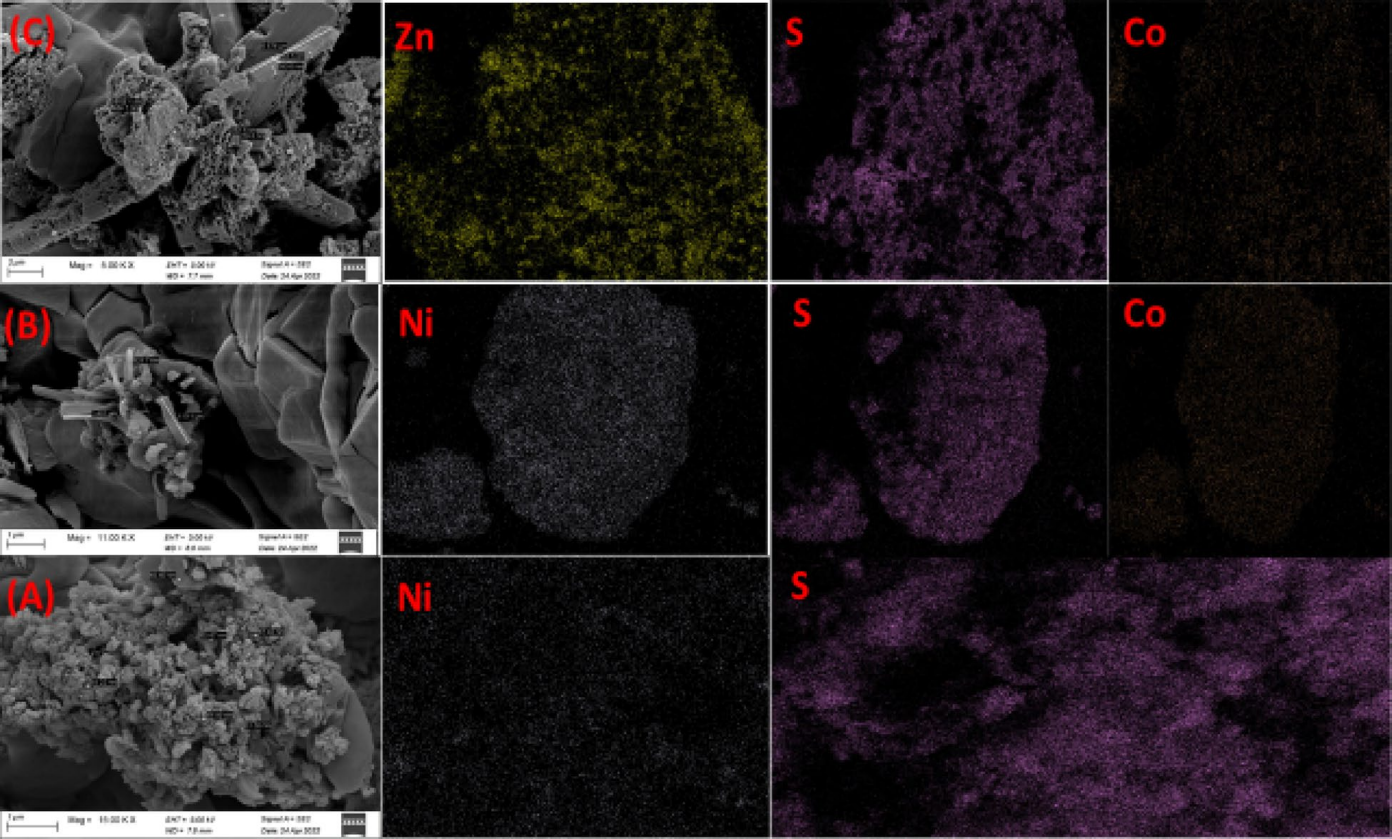
FE-SEM of (**A**) NiS, (**B**) CoNi_2_S_4_ and (**C**) Zn_0.76_Co_0.24_S nanostructures.

**Fig. 7:**
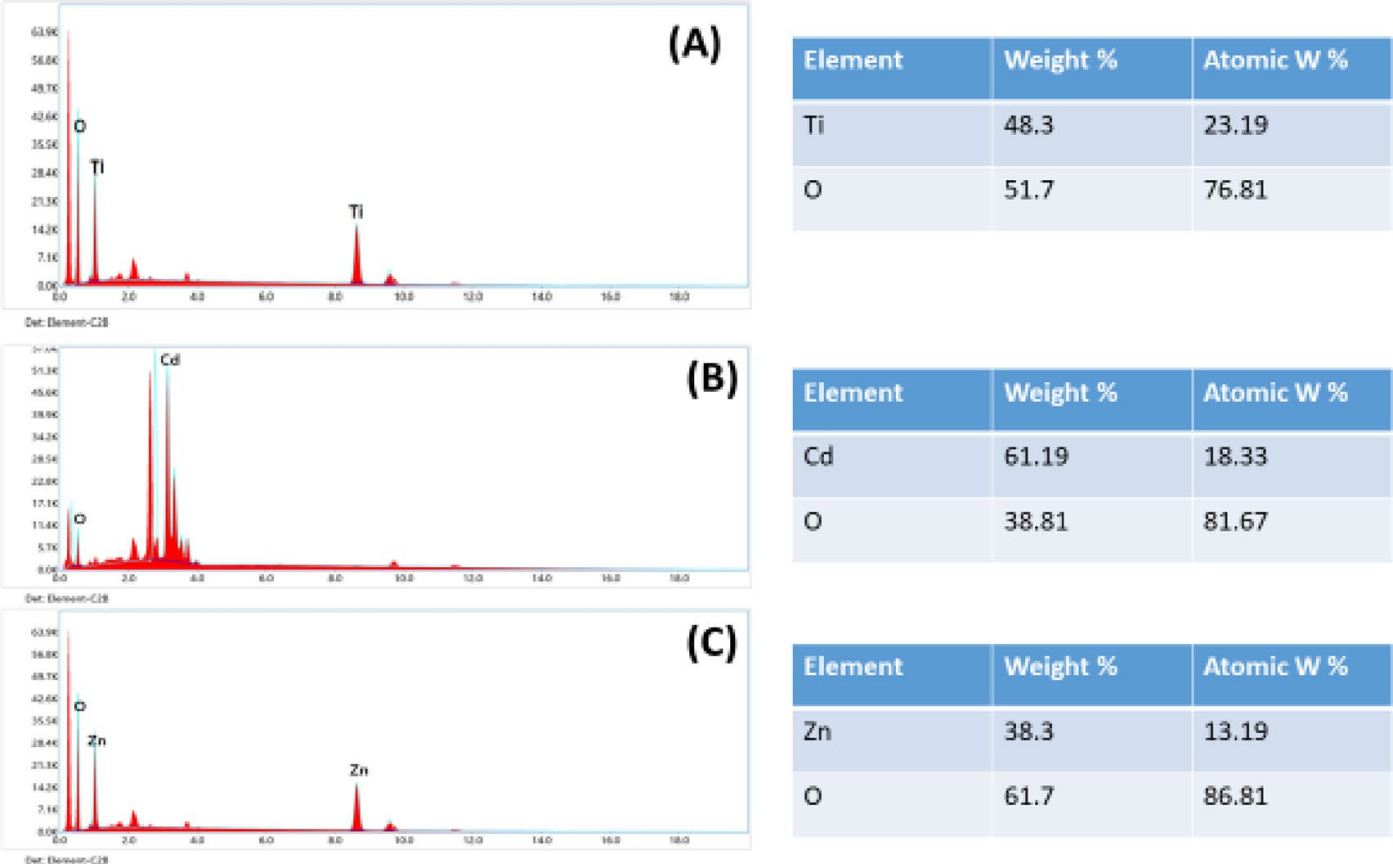
EDX of (**A**) TiO_2_, (**B**) CdO and (**C**) ZnO nanostructures.

**Fig. 8:**
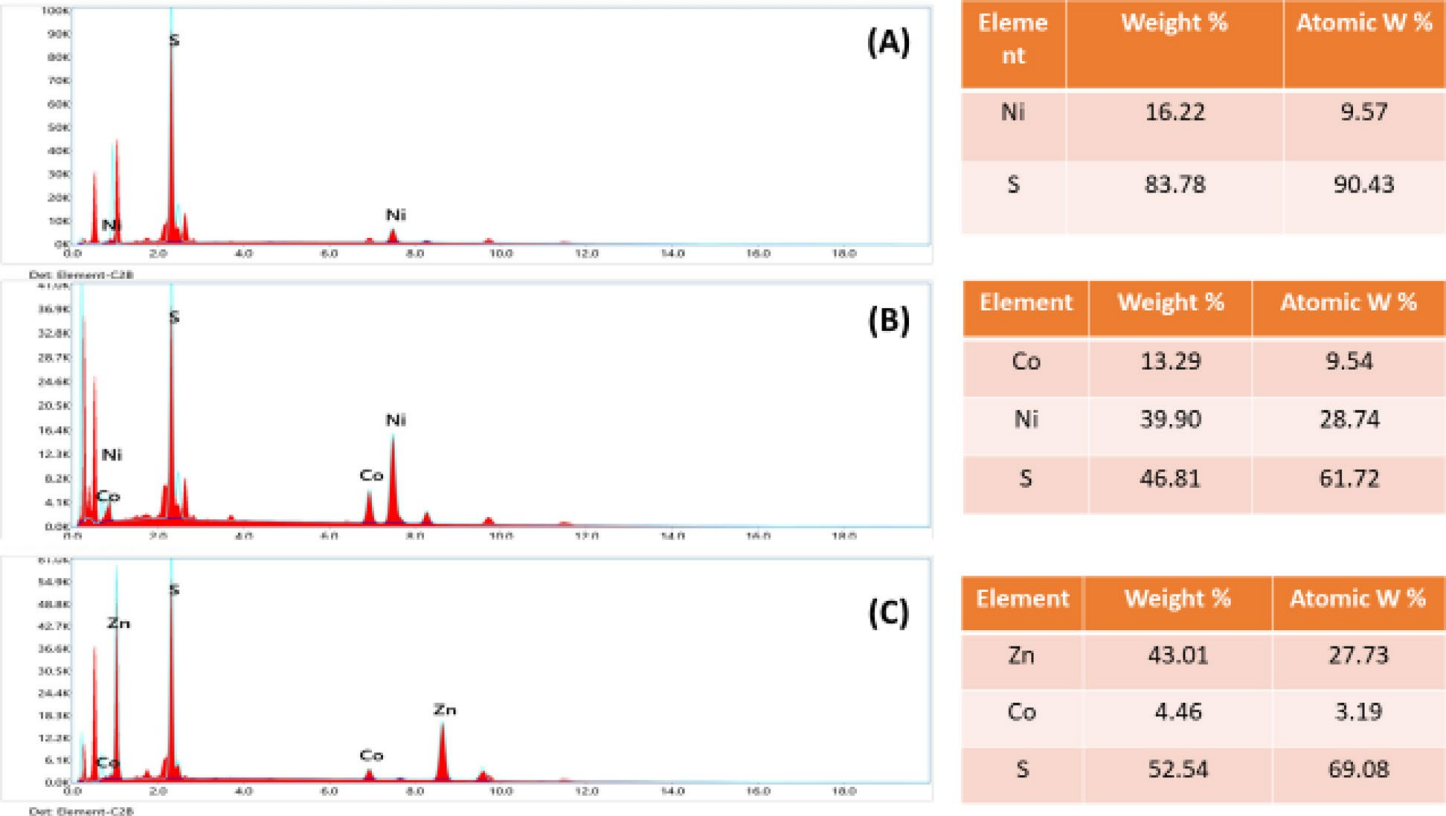
EDX of (**A**) NiS, (**B**) CoNi_2_S_4_ and (**C**) Zn_0.76_Co_0.24_S nanostructures.

The ZnO nanoparticles are consistently aligned vertically, as seen in Fig. 5B, and their average diameter is approximately 55.6 nm. It is evident that the top of ZnO has a hexagonal form, which is in line with the findings of XRD. The existence of Ti, Cd, and Zn with oxygen is confirmed by energy dispersive X-ray analysis (EDX) of the TiO2, CdO, and ZnO powder annealed at 400 °C and 600 °C, as shown in Fig. 5A–C. The FESEM images of NiS nanostructures with nano-columns arranged in a hexagonal microcage-like morphology are displayed in Fig. 6A. It is evident that the cage-like structure’s intermediate pores, which have an average diameter of 68.45 nm, are beneficial for increased electrolyte diffusion. The energy dispersive X-ray analysis (EDX) of the NiS powder, which verifies the presence of Ni and S, is displayed in Fig. 7A. The average diameter of the flakes in the FESEM images of the hydrothermally grown CoNi_2_S_4_ nanostructures (Fig. 6B) is 130.2 nm, and they are coated with a wispy lichen-like appearance. The emission peaks in the CoNi_2_S_4_ EDX spectrum, as displayed in Fig. 6B, are associated with the components Co, Ni, and S. The Zn_0.76_Co_0.24_S FESEM images in Fig. 8C show microspheres coated in tiny nanoparticles. The emission peaks in the Zn_0.76_Co_0.24_S EDX spectrum, as seen in Fig. 6C, correspond to the elements Zn, Co, and S.

Lastly, Figs. 7 and 8 show the uniform distribution of (Cd and O elements) for CdO, (Zn and O elements) for ZnO, (Ni and S elements) for NiS, (Co, Ni and S elements) for CoNi_2_S_4_, and (Zn, Co, and S elements) for Zn_0.76_Co_0.24_S. These X-ray elemental mappings of CdO, ZnO, NiS, and Zn_0_._76_Co_0.24_S.

### Optical properties

**Fig. 9:**
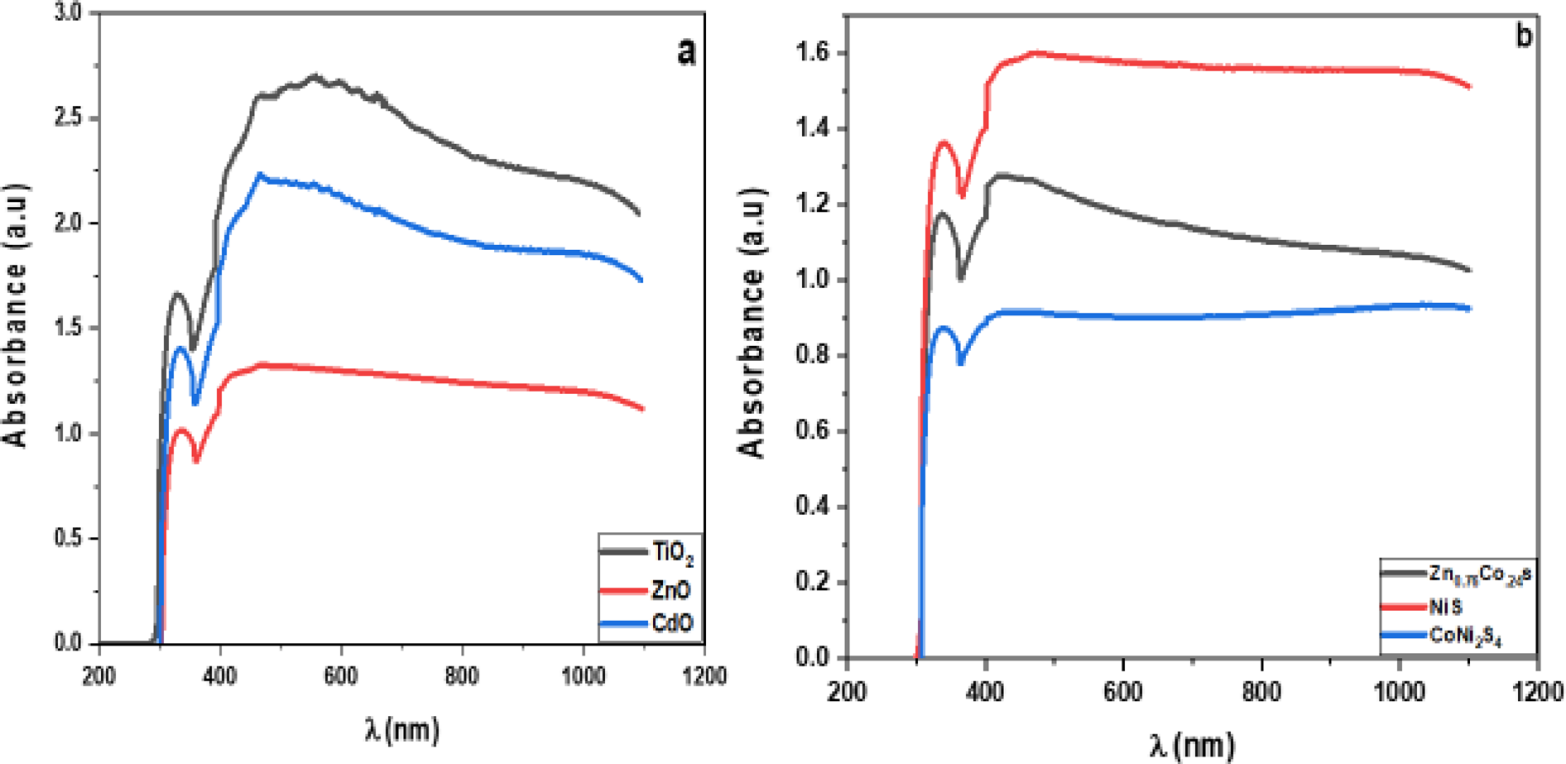
Absorbance spectra of (**a**) TiO_2_, CdO and ZnO NP_s_, (**b**) NiS, CoNi_2_S_4_ and Zn_0.76_Co_0.24_S NC_s_.

**Fig. 10:**
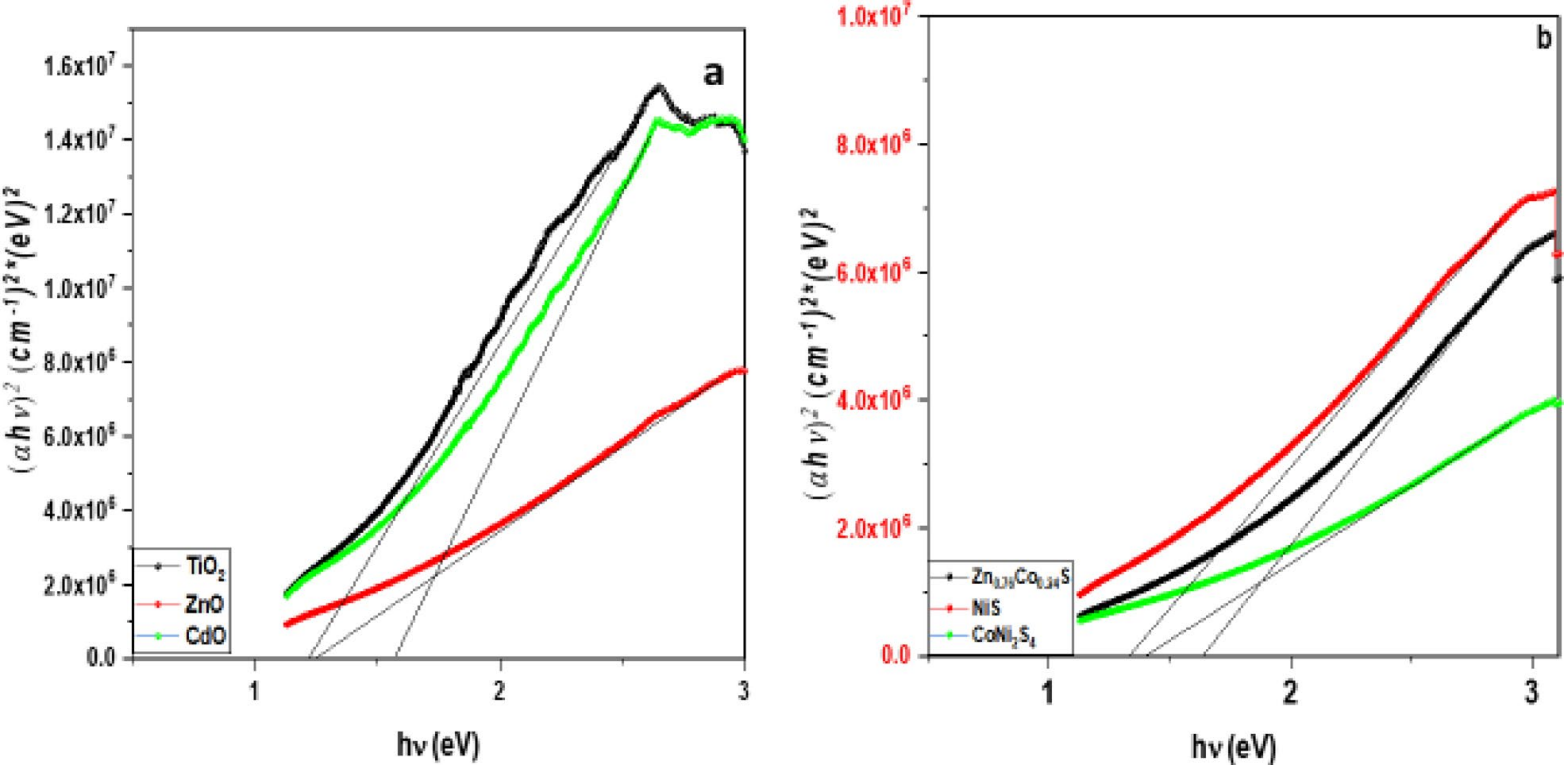
Energy gap of (**a**) TiO_2_, CdO and ZnO NP_s_, (**b**) NiS, CoNi_2_S_4_ and Zn_0.76_Co_0.24_S NC_s_.

The optical absorption spectra of ZnO, TiO_2_, and CdO nanostructures exposed to UV–Vis light are shown in Fig. 9. Optical absorption is higher in TiO_2_ NPs than in ZnO and CdO nanostructures. Figure 10 shows the absorption spectra of Zn_0.76_Co_0.24_S, NiS, and CoNi_2_S_4_ nanocomposites. The NiS nanoparticle sample has a higher absorbance than Zn_0.76_Co_0.24_S and CoNi_2_S_4_. To determine the optical band gap, we used the Tauc formula, which is as follows: (αhv) = A (hυ - Eg) n, where hυ is the photon energy, Eg is the energy gap, and n is the quality of the transitions. In the direct transition, the term n is used as 2, whereas for an indirect transition, it is ½. Plotting (αhυ) ^1/n^ versus photon energy and tangent-drawing the curve that meets the energy axis at α = 0 are the methods used to examine the optical bandgap energy.

The direct permitted transition, Fig. 9 displays the Tauc plot of pure CdO, TiO_2_, ZnO, NiS, CoNi_2_S_4_, and Zn_0.76_Co_0.24_S nanocomposites. The calculated energy gaps of CdO, TiO_2_, and ZnO nanoparticles are 1.57 eV, 1.22 eV, and 1.25 eV, respectively. Zn_0.76_Co_0.24_S, CoNi_2_S_4_, and NiS nanocomposite graphs. Figure 9a displays the Tauc plot for pure nanocomposites of CdO, TiO_2_, ZnO, NiS, CoNi_2_S_4_, and Zn_0.76_Co_0.24_S, which have a direct allowed transition. Figure 10 shows sites in direct permitted transition, with estimated energy band gaps 1.33 eV, 1.4 eV, and 1.64 eV for Zn_0.76_Co_0.24_S, CoNi_2_S_4_, and NiS nanocomposites, respectively.

### Photovoltaic performance

**Fig. 11:**
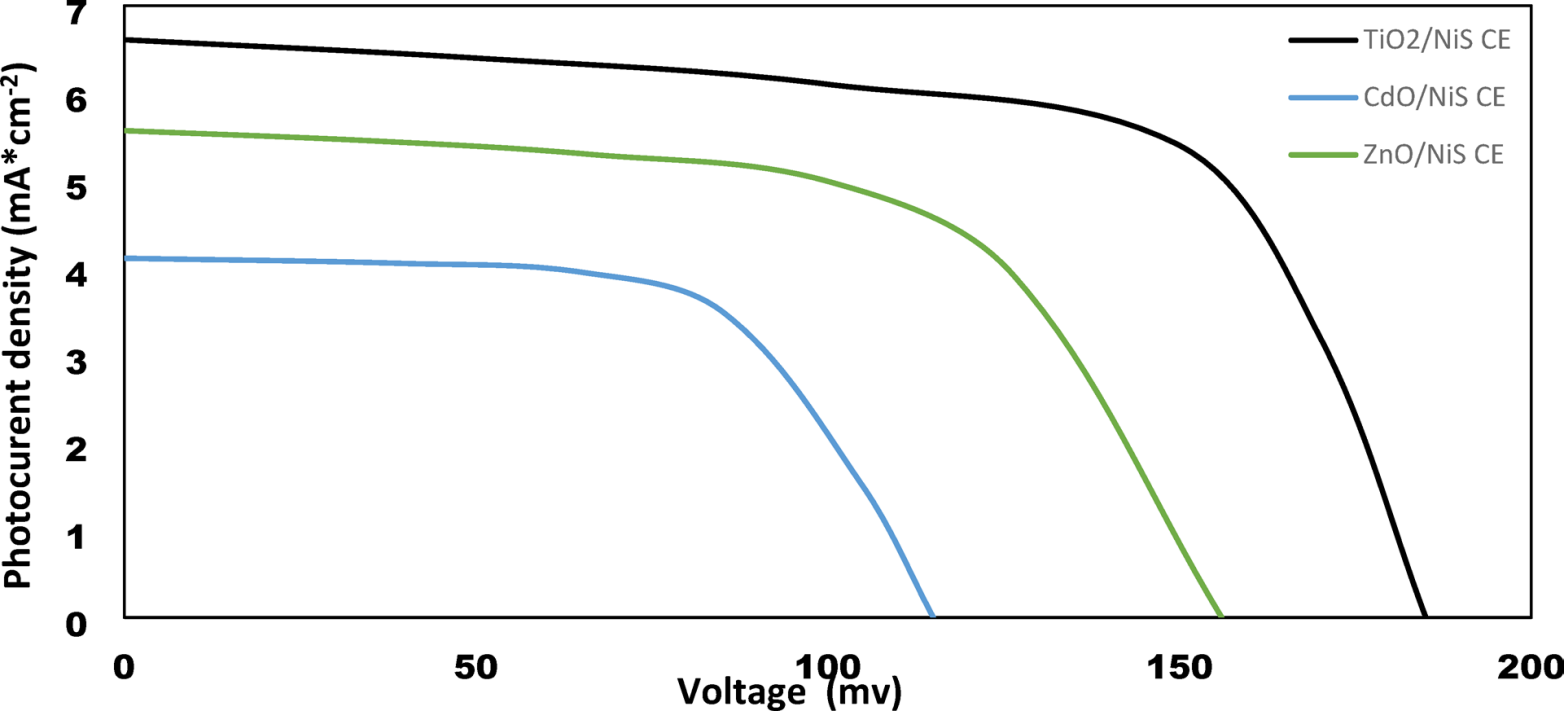
J-V curves of TiO_2_, CdO and ZnO NP_s_ electrodes based on hybrid structure of CdS/ZnS QD_s_ and NiS CE under one sun illumination (100 mWcm^− 2^).

**Fig. 12:**
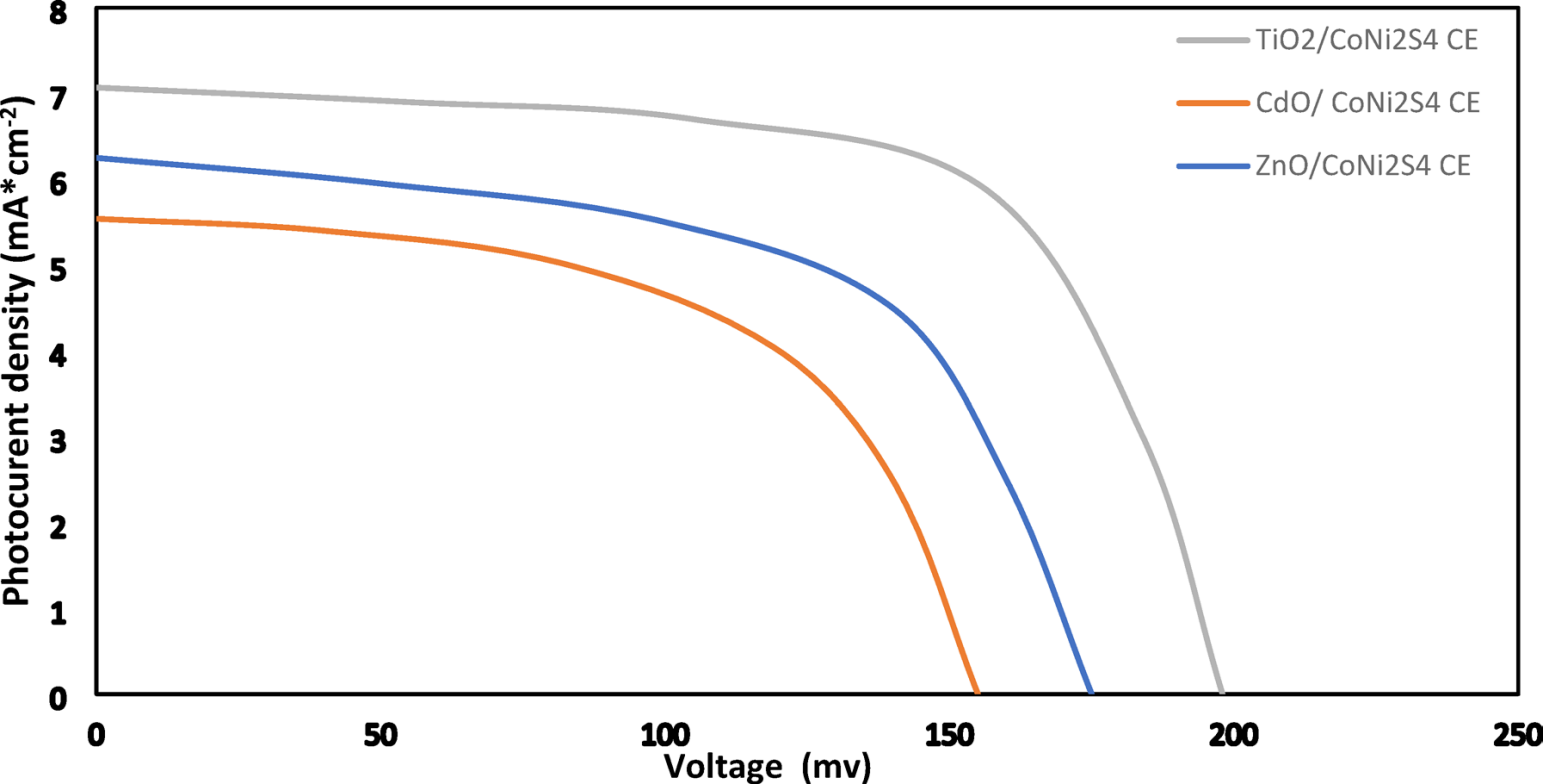
J-V curves of TiO_2_, CdO and ZnO NP_s_ electrodes based on hybrid structure of CdS/ZnS QD_s_ and CoNi_2_S_4_ CE under one sun illumination (100 mWcm^− 2^).

**Fig. 13:**
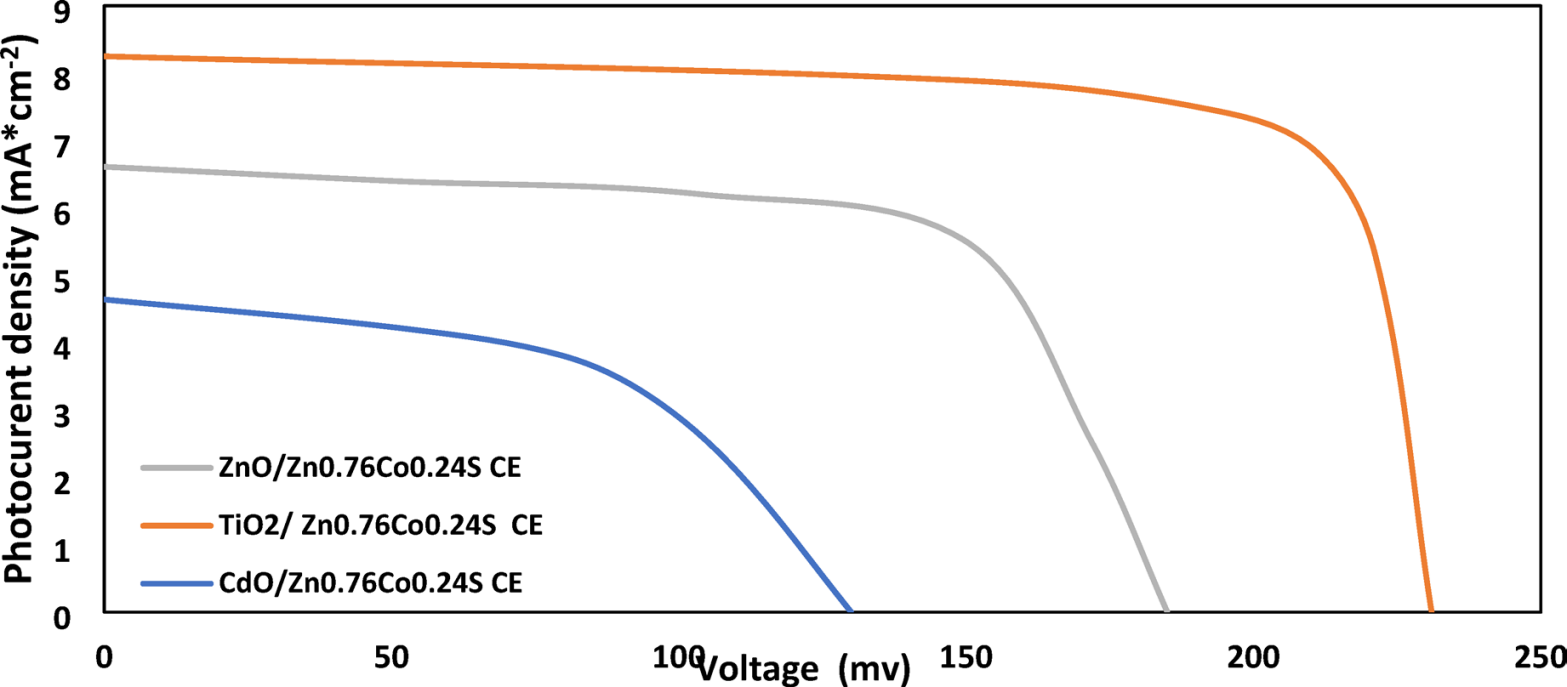
J-V curves of TiO_2_, CdO and ZnO NP_s_ electrodes based on hybrid structure of CdS/ZnS QD_s_ and Zn_0.76_Co_0.24_S NC_s_ CE under one sun illumination (100 mWcm^− 2^).

**Fig. 14:**
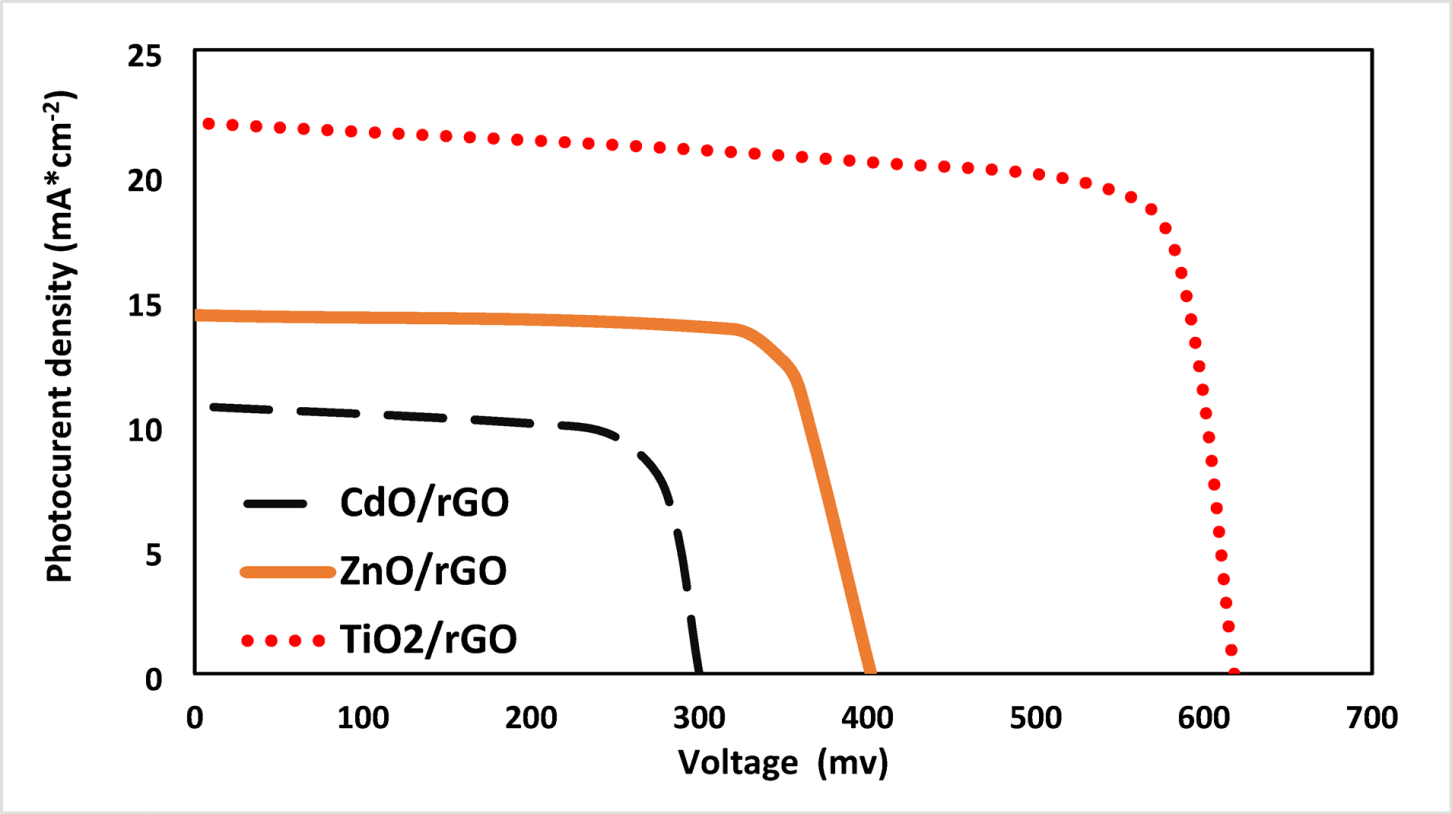
J-V curves of TiO_2_, CdO and ZnO NP_s_ electrodes based on CdS/ZnS QD_s_ and P-rGO CE under one sun illumination.

Under one sun illumination (100 mW*cm^− 2^), the J-V characteristics of photovoltaic performance of prepared systems of Hybrid QDSSCs are displayed in Figs. 11, 12, 13 and 14. These systems are composed of TiO_2_, CdO, and ZnO nanoparticles as a photoanode with hybrid structures of CdS/ZnS QD_s_ as a photosensitized nanomaterial and NiS, CoNi_2_S_4_, Zn_0.76_Co_0.24_S, and P-rGO as a counter electrode. The photovoltaic parameters J_sc_, V_oc_, FF, η%, and cell design are also summarized in Table 1. In addition, the prepared one system cell of each type and measured the photovoltaic parameters through three days then taking the average of the resultant values with calculating standered errors that shown in Table 1. With six layers of CdS QDs and six layers of ZnS QDs, the power conversion efficiency of systems that contain TiO_2_ QDs as a photoanode with P-rGO (10.75%), NiS (1.03%), CoNi_2_S_4_ (0.87%), and Zn_0.76_Co_0.24_S (1.58%) is higher than systems that contains CdO NP_s_ and ZnO NP_s_ as a photoanodes with the same above counter elctrodes. This is due to TiO_2_ QD_s_ having a high mechanical and chemical stability, their excellent separation of a wide band gap to absorbs a wide range of incident light, and transfer of light-generated electrons and holes. In addition to the basic factor that is responsible for increasing the power conversion efficiency in the system of TiO_2_ QD_s_ as a photoanode, TiO_2_ QD_s_ has a lower recombination resistance, lower impedance to increase electric current, lower diffusion rate and a higher relaxation time to be stable under irradiation light.

**Table 1:** Photovoltaic parameter of TiO_2_, CdO and ZnO with hybrid structure of (6 cycles cds/6 cycles of ZnS QD_s_) as a photosensitizer with P-rGO, Zn_0.76_Co_0.24_S, CoNi_2_S_4_ and NiS as a counter electrode.

Photoanode (PA)	Counter Electrode (CE)	J_sc_ (mA*cm^− 2^) (J_sc_ ±0.855)	V_oc_ (Volt) (V_oc_±0.007)	FF (FF ± 1)	$\:\varvec{\eta\:}$ (%) ($\:\varvec{\eta\:}$ ±0.006)
TiO_2_	P-rGO	22.07	0.618	0.82	10.75
	Zn_0.76_Co_0.24_S	8.25	0.231	0.83	1.58
	CoNi_2_S_4_	7.07	0.198	0.62	0.87
	NiS	6.61	0.185	0.84	1.03
ZnO	P-rGO	14.36	0.402	0.76	4.22
	Zn_0.76_Co_0.24_S	6.61	0.185	0.66	0.81
	CoNi_2_S_4_	6.25	0.175	0.64	0.7
	NiS	5.57	0.156	0.41	0.36
CdO	P-rGO	10.71	0.300	0.69	2.13
	Zn_0.76_Co_0.24_S	4.64	0.130	0.53	0.32
	CoNi_2_S_4_	5.54	0.155	0.60	0.52
	NiS	4.11	0.115	0.32	0.20

### Impedance spectroscopy

**Fig. 15:**
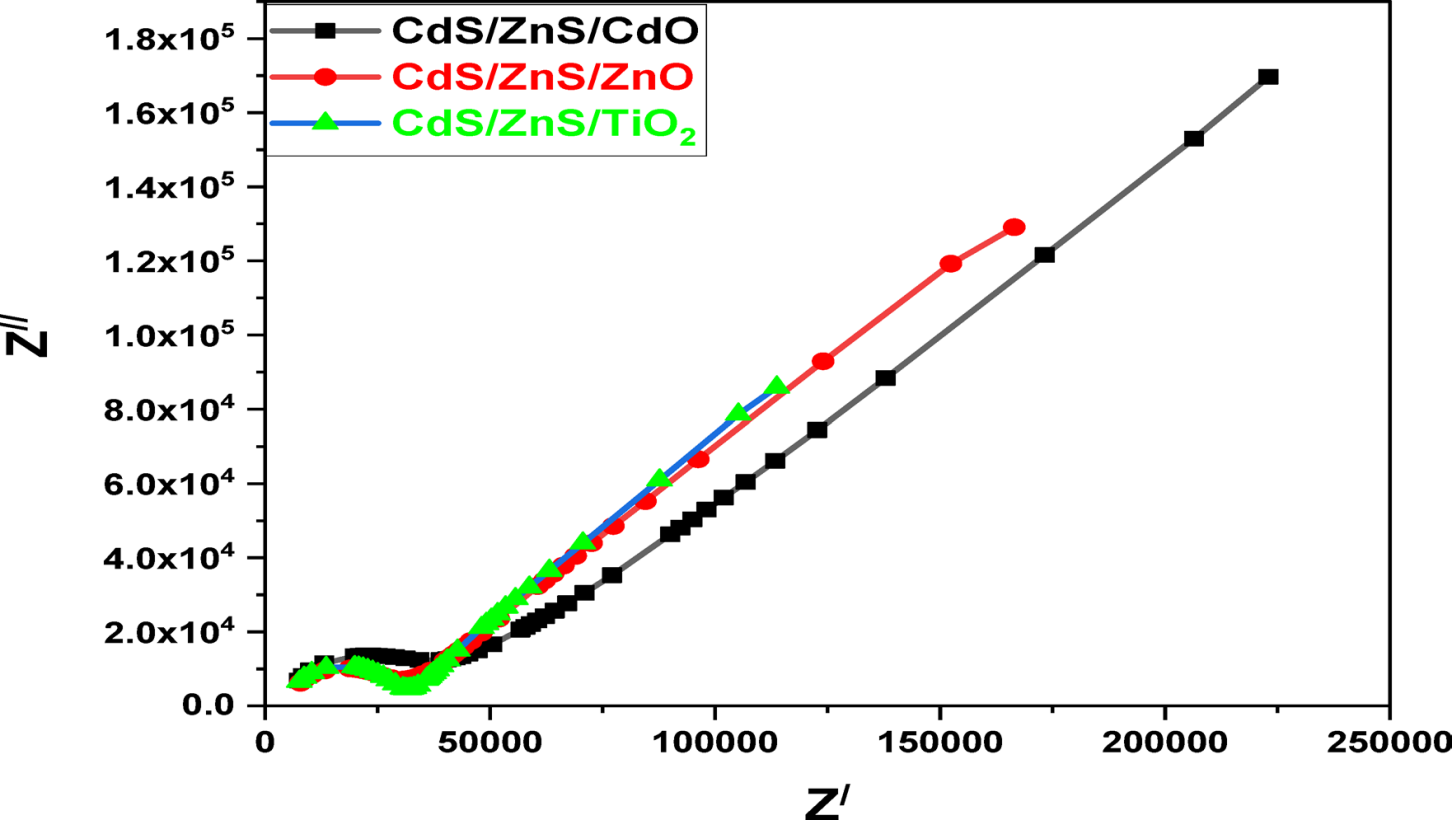
Nyquist plot of EIS spectra of CdS/ZnS QD_s_ deposited on TiO_2_ QD_s_, ZnO and CdO NPs as a photoanodes.

Electrochemical impedance spectroscopy (EIS) was used to evaluate the impedance of TiO_2_ NPs, ZnO NPs, and CdO NPs with CdS QDs on FTO conductive glass through the dark field. This information can be used to explain the kinetic operation of charge relocation in QDSSCs. Figure 15 displays one semicircle with a straight diffusion line due to the CdS/ZnS quantum dots in each sample’s Nyquist plot.

The charge transport resistance (R_ct_) for the first semicircle arc and the charge recombination resistance (R_rec_) for the second semicircle arc for high and low frequencies, respectively. A lower R_ct_ value indicates a better bond adhesion between TiO_2_ nanostructures and the FTO conductive glass substrate, supporting a higher number of electrons from the external circuit. The main reason that charge transfer resistance matters is because it makes it easier for electrons to carry during an electrolyte’s catalytic reduction process.

Lower R_ct_ values result in higher electron mobility rates, produce higher electrical outputs, and vice versa. Table 2 shows that TiO_2_ electrode (6.72*10^2^ Ω cm^2^) has a lower R_ct_ value, relaxation periods, and diffusion rates than the ZnO electrode (8.59*10^2^ Ω.cm^2^) and the CdO electrode (6.6*10^5^ Ω cm^2^) containing CdS/ZnS QDs ^61,62^. Due to the strong electrocatalytic attitude of TiO_2_, it can serve as an effective WEs catalyst for reducing the amount of oxidized polysulfide electrolyte and providing the high electron mobility rate necessary for optimal QDSSC_s_ photovoltaic performance, as the collected evidence suggests. From the subsequent formula, Eq. (4) one can compute the electron diffusion rate and the lifetimes (relaxation times) of photogenerated electrons (τ_n_) from Eq. 3.

**Table 2:** Electrochemical impedance of TiO_2_ QD_s_, ZnO and CdO with 12 cycles cds/zns QD_s_ as photoanodes.

Sample	*R*_ct_ Ω cm^2^	*R*_rec_ Ω cm^2^	C_a_, Farad (F)	Z_f_ (Ω cm^2)^	τ_*n* (ms)_	K_eff_ (ms)^−1^
TiO_2_	6.72*10^2^	6.88*10^2^	2.88*10^–14^	1.72*10^2^	4.35*10^− 3^	22.05
ZnO	8.59*10^2^	31.8*10^4^	1.34*10^–14^	1.09*10^4^	2.7*10^− 3^	37.03
CdO	6.6*10^5^	24.92*10^5^	8.11*10^–16^	1.26*10^4^	1.85*10^− 3^	54.05

## Conclusion

Using the SILAR technique, CdS/ZnS/ZnO, CdS/ZnS/TiO_2_, and CdS/ZnS/CdO were successfully coated on the FTO substrate. The highly electrocatalytically active CoNi_2_S_4_, Zn_0.76_Co_0.24_S, NiS and P-rGO CE_s_ were produced using the doctor blade method. The structural and morphological characteristics and the electrical properties of photoanodes and counter electrodes were investigated.

The most effective system of the prepared hybrid quantum dot sensitized solar cells was the CdS/ZnS/TiO_2_ cell based on a P-rGO counter electrode, which had an efficiency of 10.75%. The system CdS/ZnS/TiO_2_ cell based on a Zn_0.76_Co_0.24_S counter electrode had an efficiency of 1.58%,  while the efficiency of the CdS/ZnS/TiO_2_ cell based on a NiS counter electrode had an efficiency of 1.03%. The energy barrier at the interface between the photoanode and counter electrodes lowers the rate of charge recombination, leading to this enhancement in QDSSC efficiency.

## Data Availability

All data generated or analysed during this study are included in this published article.
